# Poly(Ethylene Glycol)-*b*-Poly(D,L-Lactide) Nanoparticles as Potential Carriers for Anticancer Drug Oxaliplatin

**DOI:** 10.3390/molecules26030602

**Published:** 2021-01-24

**Authors:** Yulia A. Kadina, Ekaterina V. Razuvaeva, Dmitry R. Streltsov, Nikita G. Sedush, Eleonora V. Shtykova, Alevtina I. Kulebyakina, Alexander A. Puchkov, Dmitry S. Volkov, Alexey A. Nazarov, Sergei N. Chvalun

**Affiliations:** 1 National Research Center “Kurchatov Institute”, 123182 Moscow, Russia; yellow_jk@mail.ru (Y.A.K.); streltsov.dmitry@gmail.com (D.R.S.); nsedush@gmail.com (N.G.S.); alya.kulebyakina@gmail.com (A.I.K.); puchkov1208@gmail.com (A.A.P.); s-chvalun@yandex.ru (S.N.C.); 2 Federal Scientific Research Centre “Crystallography and Photonics” of Russian Academy of Sciences, 119333 Moscow, Russia; viwopisx@yahoo.co.uk; 3 Department of Chemistry, Lomonosov Moscow State University, 119991 Moscow, Russia; dmsvolkov@gmail.com (D.S.V.); nazarov@med.chem.msu.ru (A.A.N.); 4 Enikolopov Institute of Synthetic Polymeric Materials Russian Academy of Sciences, 117393 Moscow, Russia

**Keywords:** poly(lactide), poly(ethylene glycol), block copolymers, self-assembly, nanoparticles, drug delivery systems, anticancer agent

## Abstract

Nanoparticles based on biocompatible methoxy poly(ethylene glycol)-*b*-poly(D,L-lactide) (mPEG_113_-*b*-P(D,L)LA*_n_*) copolymers as potential vehicles for the anticancer agent oxaliplatin were prepared by a nanoprecipitation technique. It was demonstrated that an increase in the hydrophobic PLA block length from 62 to 173 monomer units leads to an increase of the size of nanoparticles from 32 to 56 nm. Small-angle X-ray scattering studies confirmed the “core-corona” structure of mPEG_113_-*b*-P(D,L)LA*_n_* nanoparticles and oxaliplatin loading. It was suggested that hydrophilic oxaliplatin is adsorbed on the core-corona interface of the nanoparticles during the nanoprecipitation process. The oxaliplatin loading content decreased from 3.8 to 1.5% wt./wt. (with initial loading of 5% wt./wt.) with increasing PLA block length. Thus, the highest loading content of the anticancer drug oxaliplatin with its encapsulation efficiency of 76% in mPEG_113_-*b*-P(D,L)LA*_n_* nanoparticles can be achieved for block copolymer with short hydrophobic block.

## 1. Introduction

In the last decades, great attention has been paid to the development of nanoscale vehicles for drug delivery [1,2,3]. The incorporation of drug molecules into nanocarriers allows us to overcome poor water solubility of hydrophobic drugs, as well as increase stability against hydrolytic degradation of hydrophilic ones [4]. Moreover, nanoparticulate drug formulations can act in a targeted and prolonged manner, enhancing the efficacy of treatment, e.g., cancer treatment [4]. Platinum-based complexes (cisplatin, carboplatin, oxaliplatin, etc.) are widely used chemotherapeutics agents for the treatment of various types of cancer [5,6]. Cisplatin (*cis*-(eblock)dichloridoplatinum(II)) is the first-generation platinum drug, that has a therapeutic effect against breast cancer, ovarian cancer, lung and head and neck cancer, cervix carcinoma, etc. However, it produces significant side effects such as ototoxicity, hematological, and emetogenicity [7]. Carboplatin (cis-diammine(1,1-cyclobutanedicarboxylato)platinum(II)), that is the second-generation platinum complex and cisplatin analogue, was designed to reduce the dose limiting toxicity of cisplatin. Oxaliplatin ((*trans*-R,R-cyclohexane-1,2-diamine)oxalatoplatinum(II)) is the third-generation platinum complex that was designed to overcome cellular resistance to cisplatin and carboplatin. Oxaliplatin shows higher solubility and less toxicity than cisplatin. It is used as a standard treatment for colorectal cancer. Moreover, oxaliplatin can be active against refractory ovarian cancer, germ-cell cancers, non-small cell lung cancer, etc. Nevertheless, its low water solubility, short half-life in the bloodstream, and non-selective biodistribution reduces the effective dose of oxaliplatin in the targeted tissues and enhances the systemic toxicity [8].

Design of nanocarriers for oxaliplatin delivery is one of the strategies to overcome its limitations and improve the efficacy of cancer treatment [7,8,9]. A wide range of various types of nanocarriers including inorganic nanoparticles [10], dendrimers [11], liposomes [12], polymeric nanoparticles [13], block-copolymer micelles [14,15], nanogels [16], etc., have been investigated for drug delivery of oxaliplatin. Several liposomal formulations of oxaliplatin, e.g., Lipoxal (Regulon, Inc.) and MBP-426 (Mebiopharm Co., Ltd.), are under clinical investigation [12]. Lipoxal is based on oxaliplatin-loaded PEGylated liposomes prepared from soy phosphatidylcholine, cholesterol, dipalmitoyl phosphatidylglycerol, and mPEG-distearoyl phosphatidylethanolamine, which exhibits reduced side effects compared to free oxaliplatin [17]. MBP-426 is a transferrin (Tf)-conjugated N-glutaryl phosphatidylethanolamine liposomal formulation of oxaliplatin, which can provide selective tumor targeting by binding to transferrin receptors [18]. Nonetheless, this technology has some disadvantages, namely rapid leakage of the incorporated drug molecules from liposomes during storage and after administration [19]. The promising nanoformulations of oxaliplatin are based on various polymeric carriers. The strategies of oxaliplatin incorporation into polymeric vehicles are generally based on chemical conjugation (complexation) or physical encapsulation. Chemical conjugation is usually accomplished between block copolymers, containing poly(glutamic acid) Pglu [20,21,22], poly(methacrylic acid) (PMA) [14], or poly(lactide-*co*-2-methyl-2-carboxyl-propylene carbonate) P(LA-*co*-MCC) [23,24] block and active part of oxaliplatin dichloro(1,2-diaminocyclohexane)platinum(II) DACHPt. Cabral et al. prepared DACHPt-loaded poly(ethylene glycol)-*b*-Pglu PEG-*b*-Pglu micelles, consisting of Pglu core surrounded by PEG corona, through polymer-metal complex formation of DACHPt with Pglu block [20,21]. It was observed that an increase of the initial [DACHPt]/[Glu] molar ratio from 0.25 to 1.5 leads to enhancement of platinum encapsulation efficiency from 20% to 90% and a growth of hydrodynamic diameter of DACHPt-loaded micelles from 25 to 50 nm [20]. In ref. [21], the effect of PEG_272_-*b*-Pglu*_n_* (*n* = 20, 40, 70) copolymer composition on the DACHPt loading efficacy and biodistribution in vivo of DACHPt-loaded micelles was studied. It was observed that incorporation efficacy of DACHPt was approximately 30%, regardless of Pglu block length (molar ratio [DACHPt]/[Pglu] = 1). Meanwhile, in vivo biodistribution assay performed on tumor-bearing mice showed that DACHPt-loaded micelles based on PEG_272_-*b*-Pglu_20_ copolymer with the shortest Pglu block exhibited the lowest non-specific accumulation in normal tissues (liver, kidney, spleen) providing the highest accumulation in tumor. The authors suggested that PEG_272_-*b*-Pglu_20_ copolymer allows the formation of particles with effective surface coverage by PEG leading to reduction of accumulation of particles in liver. Although chemical conjugation usually leads to higher drug loading content in micelles, polymers can coordinate with Pt-complexes in a non-specific geometry, resulting in uncontrolled crosslinking [25]. Physical encapsulation of oxaliplatin into polymeric carriers allows us to overcome this limitation. Cui et al. developed poly(lactide) PLA nanoparticles stabilized by Tween80 as potential carriers for oxaliplatin [13]. The authors studied the effect of formulation parameters on the size, stability, drug loading content, and encapsulation efficacy of the nanoparticles. Optimal oxaliplatin loading content and its encapsulation efficacy was found to be 3.52 ± 0.07 wt.% and 17.40 ± 0.47 %, correspondingly. Micelles based on a stearic acid-grafted chitosan oligosaccharide CSO-SA were also investigated as carriers for oxaliplatin [26]. The highest drug loading content in CSO-SA micelles was 3.5 wt.%, and its encapsulation efficiency was 47%. The authors reported that a chitosan-based nanoformulation of oxaliplatin demonstrates enhanced antitumor activity in vitro against several cancer cells (about 3–6 folds) compared to free oxaliplatin. Despite a number of nanoformulations developed using different platforms, none of them have received FDA approval at the moment. The design of new types of nanocarriers for delivery of oxaliplatin is of high interest. We believe that PLA-*b*-PEG nanoparticles are a promising platform due to their flexibility and successful track record as nanocarriers for development of targeted anticancer drug formulations.

Nanoparticles of amphiphilic block copolymers comprising PLA hydrophobic block and PEG hydrophilic block have been extensively studied as potential vehicles for drug delivery due to their biocompatibility and biodegradability [27,28,29]. In aqueous solution, PLA-*b*-PEG copolymers are able to self-organize into “core-corona” nanosized structures, where hydrophobic PLA block chains form an inner core surrounded by a corona of hydrophilic PEG block chains [27,28]. PEG chains provide a “stealth effect” for the nanoparticles, minimize their undesirable interaction with proteins and capturing by the reticuloendothelial system, that extends circulation time of the nanoparticles in the body [4]. The nanoscale size of these block copolymer particles is favorable for passive targeting due to the so-called enhanced permeability and retention (EPR) effect. It is known that tumors and inflamed tissues are characterized by increased vascular permeability and impaired lymphatic drainage, which result in selective accumulation of nanoparticles into them [30]. Depending on the characteristics of the block copolymer (composition, molecular architecture, and block lengths) as well as the preparation conditions, PLA-*b*-PEG nanoparticles of different sizes and morphologies can be obtained [30]. Garofalo et al. showed that PLA/PEG block copolymer architecture, as well as its chemical composition, strongly affect size, stability, and tumor uptake of the micelles [27]. Linear and “tree-shaped” block copolymers mPEG_45_-*b*-(PLA)*_n_* and mPEG_113_-*b*-(PLA)*_n_*, where *n* = 1 and 2 or 4, with various length and tacticity of the hydrophobic PLA blocks, were synthesized. It was reported that only the two-arm mPEG_45_-*b*-poly(D,L-lactide)_2_ mPEG_45_-*b*-(P(D,L)LA)_2_ copolymer produces high-stable monodispersed micelles with a hydrodynamic diameter of about 250 nm, whereas the other block copolymers show low stability and tendency to aggregate with formation of large submicron clusters. The influence of hydrophobic block length on the size of P(D,L)LA-*b*-PEG nanoparticles was studied in ref. [31]. It was reported that the hydrodynamic diameter of P(D,L)LA-*b*-PEG_113_ nanoparticles produced by nanoprecipitation increased from 27.7 to 174.6 nm, with an increase of P(D,L)LA block molecular weight from 3 to 110 kDa. The effect of the nanoprecipitation parameters on the size of mPEG_113_-*b*-P(D,L)LA_800_ nanoparticles was studied by Y. Dong and S.-S. Feng [32]. The increase of the polymer concentration in organic phase from 4 to 13 g/L leads to increasing hydrodynamic diameter of the particles from 77 to 111.2 nm. Meanwhile, the increase of the organic solvent volume from 5 to 25 mL with a fixed polymer concentration of 10 g/L results in reducing hydrodynamic diameter of the particles from 89.8 to 79.7 nm.

For application of block copolymer nanoparticles as carriers for drug delivery, considerable attention should be given to their structure. Small-angle X-ray (SAXS) and neutron (SANS) scattering are powerful techniques for characterization of nanostructure, which can be implemented to analyze micelles and nanoparticles in aqueous medium [33]. Important characteristics affecting physicochemical properties of core-corona nanoparticles, and their drug loading capability can be determined from small-angle scattering data, e.g., density and size of the core, thickness and surface density of the corona, etc. [34,35,36,37,38]. Using SANS, T. Riley et al. studied the structure of deuterated P(D,L)LA(d)-*b*-PEG nanoparticles in aqueous solution, prepared by solvent evaporation method [36]. It was found that the P(D,L)LA(d) block length affects the core size as well as conformation of the corona-forming PEG chains. Thus, P(D,L)LA(d)-*b*-PEG_113_ with short P(D,L)LA block (3 kDa) forms nanoparticles with a small core and highly splayed PEG chains in corona. The increase of the P(D,L)LA molecular weight to 15 kDa at a fixed length of the PEG block leads to increasing core size and, consequently, reducing PEG chain grafting density. The authors also observed that with decreasing PEG chain grafting density, the corona becomes more radially homogeneous. Using SAXS, Ma et al. investigated the influence of PLA stereostructure on the density of core and doxorubicin loading efficacy of PLA/PEG nanoparticles, which were prepared by a precipitation/solvent evaporation method [37]. It was observed that nanoparticles formed of enantiomerically-mixed P(L)LA_64_-*b*-PEG_113_/P(D)LA_71_-*b*-PEG_113_ copolymers and P(L)LA_64_-*b*-PEG_113_ copolymer exhibit larger core density compared with nanoparticles with stereoblock copolymer core, e.g., PEG_113_-*b*-P(L)LA_32_-P(D)LA_34_, and P(D,L)LA_58_-*b*-PEG_113_.

Various fabrication techniques were developed to incorporate drugs in the core of the nanoparticles or at the core-corona interface, e.g., direct dissolution, dialysis, nanoprecipitation, salting-out method, emulsification method, etc. [39]. There are many factors that affect drug loading, including drug-core compatibility [40,41,42], hydrophobic block length [31,43,44,45], its crystallization capability [37,44,45], preparation conditions [32], etc. Despite the recent progress in development of the drug nanoformulations based on nanoparticles of amphiphilic block copolymers, there is still a lack of systematic data on the relationship between the chemical structure of block copolymer and the drug loading ability of nanoparticles. X. Zhang et al. investigated nanoparticles of P(D,L)LA-*b*-mPEG copolymers with various P(D,L)LA/mPEG weight ratios prepared by a solution casting method as potential carriers of highly hydrophobic drug paclitaxel [43]. It was reported that the particles based on P(D,L)LA-*b*-mPEG copolymers with higher P(D,L)LA content exhibited enhanced paclitaxel loading efficacy. Nanoparticles prepared by a nanoprecipitation method from P(D,L)LA-*b*-PEG copolymers with a fixed molecular weight of PEG block (5 kDa) and a variable molecular weight of P(D,L)LA block (from 3 to 110 kDa) were studied as a delivery system for water-soluble drug procaine hydrochloride [31]. It was demonstrated that the drug incorporation efficacy was independent of the P(D,L)LA block molecular weight.

PEG-*b*-PLA nanoparticles demonstrate many advantages as carriers for anticancer drugs. However, to the best of our knowledge, there is only one research article dedicated to PEG-*b*-PLA nanoparticles as potential carriers of oxaliplatin [15]. In addition, there is no data on the relationship between the chemical structure of PEG-*b*-PLA copolymers and the oxaliplatin loading ability of these nanoparticles. Development of such nanoformulation is complicated due to hydrophilicity of oxaliplatin, which limits its loading into carriers. The precise control of nanoparticles’ structure and properties throughout adjustment of molecular structure of amphiphilic PEG-*b*-PLA copolymers is a promising approach for tuning their loading capacity. In the present work, we propose a nanoformulation of Pt(II)-based complex oxaliplatin based on mPEG_113_-*b*-P(D,L)LA*_n_* nanoparticles, where *n* = 62–173 monomer units. Amphiphilic block copolymers with rather short PLA blocks (< 200 monomer units) are of great interest because significant changes in structure and characteristics of nanoparticles can occur with increasing PLA block length in this range of polymerization degrees. The aim of our study was to elucidate the effect of molecular weight of the hydrophobic P(D,L)LA block on the size, structure, morphology, and drug loading of the mPEG-*b*-P(D,L)LA nanoparticles.

## 2. Results and Discussion

### 2.1. Synthesis of mPEG-b-P(D,L)LA Copolymers

**Figure 1:**
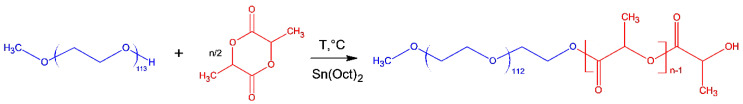
Scheme of synthesis of mPEG-*b*-P(D,L)LA copolymers by ring-opening polymerization.

Amphiphilic mPEG_113_-*b*-P(D,L)LA*_n_* copolymers with fixed molecular weight of the PEG block were synthesized by ring-opening polymerization (Figure 1). It is known that hydroxyl-containing compounds act as co-initiators in a coordination-insertion polymerization of lactide in presence of SnOct_2_ catalyst [46]. Therefore, block-copolymers can be synthesized with mPEG as a macroinitiator.

Molecular characteristics of the synthesized polymers are presented in Table 1. The residual content of monomer determined by ^1^H NMR was less than 1% for all block copolymers.

### 2.2. Characterization of Drug-Free mPEG-b-(D,L)LA Nanoparticles

**Figure 2:**
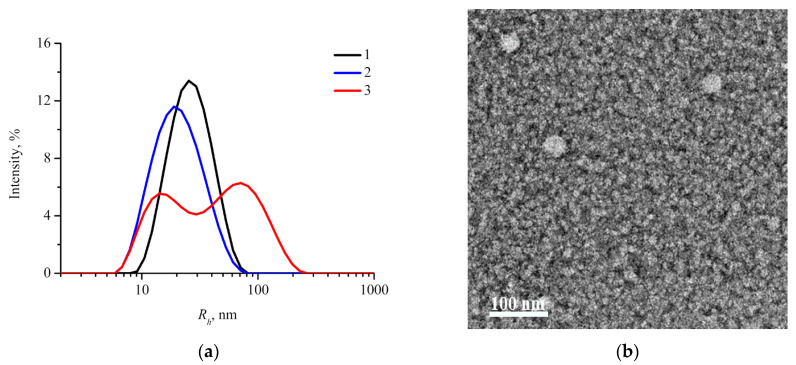
(**a**) DLS intensity size distribution curves for nanoparticles based on mPEG_113_-*b*-P(D,L)LA*_n_* (c = 0.5 g/L): 1—mPEG_113_-*b*-P(D,L)LA_173_, 2—mPEG_113_-*b*-P(D,L)LA_135_, 3—mPEG_113_-*b*-P(D,L)LA_62_; (**b**) representative TEM image of spherical individual mPEG_113_-*b*-P(D,L)LA_62_ nanoparticles.

For targeted delivery through EPR-effect hydrodynamic diameter of nanoparticles should be in the range of 10–200 nm [30]. Therefore, physicochemical characteristics of nanoparticles are important parameters that should be studied in order to consider them as potential drug carriers. Intensity size distribution curves (DLS) of aqueous suspensions of drug-free mPEG_113_-*b*-P(D,L)LA*_n_* nanoparticles are presented in Figure 2a. Monomodal distribution with hydrodynamic radius *R_h_* values corresponding to the peak maximum of 22 ± 10 and 28 ± 10 nm are observed for mPEG_113_-*b*-P(D,L)LA_135_ and mPEG_113_-*b*-P(D,L)LA_173_, respectively. A second peak appears on the intensity size distribution curve of mPEG_113_-*b*-P(D,L)LA_62_ nanoparticles with the shortest PLA block. One can suggest that these two peaks can be attributed to small individual nanoparticles and their large aggregates. For individual mPEG_113_-*b*-P(D,L)LA_62_ nanoparticles and their aggregates the values of *R_h_* corresponding to the peak position were found to be 16 ± 6 and 72 ± 40 nm, respectively. Since the light scattering intensity of large objects is much stronger than that of small objects [47] and the peak intensities are almost equal, one can assume that only a minor fraction of the aggregates co-exists with the main fraction of individual nanoparticles. This assumption was also confirmed by TEM and SAXS measurements, provided below.

Ζ-Potential of drug-free mPEG_113_-*b*-P(D,L)LA*_n_* nanoparticles was found to be in the range from −14 to −20 mV (Table 2), which promotes their high stability in water.

For all the mPEG_113_-*b*-P(D,L)LA*_n_* copolymers, TEM reveals only spherical individual nanoparticles (Figure 2b).

**Figure 3:**
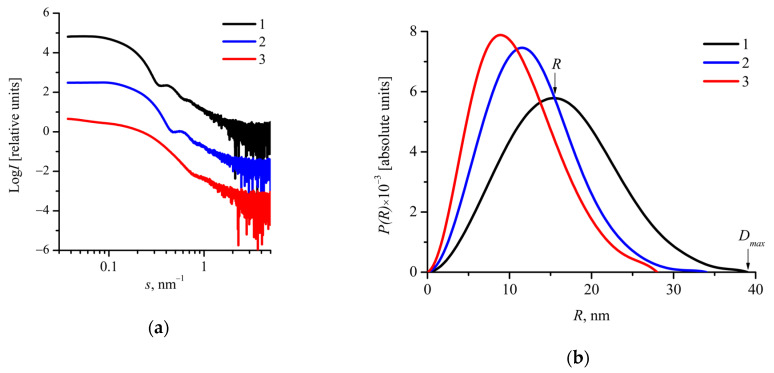
(**a**) SAXS (small-angle X-ray) curves in Log *I*–Log *s* coordinates and (**b**) corresponding pair distance distribution functions *P(R)*–*R* for mPEG_113_-*b*-P(D,L)LA*_n_* nanoparticles: 1—mPEG_113_-*b*-P(D,L)LA_173_ (c = 7.5 g/L), 2—mPEG_113_-*b*-P(D,L)LA_135_ (c = 5 g/L), 3—mPEG_113_-*b*-P(D,L)LA_62_ (c = 5 g/L). The SAXS curves are shifted vertically for clarity.

In order to evaluate the size and address the structure of nanoparticles, SAXS measurements were carried out. Scattering curves for aqueous suspensions of mPEG_113_-*b*-P(D,L)LA*_n_* nanoparticles in Log *I*–Log *s* coordinates are presented in Figure 3a. All the SAXS curves converge to a plateau in the region of *s* < 0.1 nm^−1^, indicating the absence of large scattering objects (aggregates) in aqueous suspensions of mPEG_113_-*b*-P(D,L)LA*_n_* nanoparticles [48]. Apparently, the aggregates were removed during centrifugation before SAXS measurements. One also can see the secondary maximum at the SAXS curves in the region 0.3 < *s* < 1 nm^−1^ (Figure 3a), suggesting that the nanoparticles have a spherical, well-defined structure with a relatively narrow size distribution.

**Figure 4:**
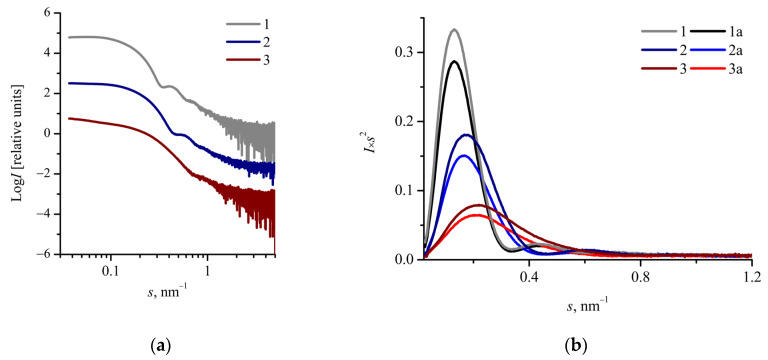
(**a**) SAXS curves in Log *I*–Log *s* coordinates for oxaliplatin-loaded mPEG_113_-*b*-P(D,L)LA*_n_* nanoparticles: 1—mPEG_113_-*b*-P(D,L)LA_173_ (c = 7.5 g/L), 2—mPEG_113_-*b*-P(D,L)LA_135_ (c = 5 g/L), 3—mPEG_113_-*b*-P(D,L)LA_62_ (c = 5 g/L). The SAXS curves are shifted vertically for clarity. (**b**) Kratky plots for oxaliplatin-loaded (1, 2, 3) and drug-free (1a, 2a, 3a) mPEG_113_-*b*-P(D,L)LA*_n_* nanoparticles: 1—mPEG_113_-*b*-P(D,L)LA_173_ (c = 7.5 g/L), 2—mPEG_113_-*b*-P(D,L)LA_135_ (c = 5 g/L), 3,—mPEG_113_-*b*-P(D,L)LA_62_ (c = 5 g/L).

The bell-shaped Kratky plots indicate a compact globular shape of the nanoparticles (Figure 4b) [49].

The values of gyration radius *R_g_* were determined from the Guinier plots (Figure S1 of the Supplementary Materials). It was found that an increase in the P(D,L)LA block length results in higher *R_g_* values (Table 2).

The pair distance distribution functions *P(R)* are presented in Figure 3b. They are bell-shaped with a peak shifted to distances smaller than a half of maximum dimension of the scattering objects *D_max_/2* (Figure 3b). Since solid spherical particles display bell-shaped *P(R)* functions with a peak at about *D_max_/2* [50], one can assume that the shift of *P(R)* function maximum could be attributed to “core-corona” structure of the mPEG_113_-*b*-P(D,L)LA*_n_* nanoparticles.

The values of *R* and *D_max_/2* corresponding to the peak position and the maximum dimension evaluated from *P(R)* functions are listed in Table 2. It was found that an increase in the P(D,L)LA block length leads to higher *R* and *D_max_/2* values. *R_g_* values evaluated from *P(R)* functions were compared with *D_max_/2* and *R* values. The *2R_g_/D_max_* values were found to be in the range of 0.57–0.64, whereas *R_g_/R* was in the range of 0.81–0.96 (Table 2). It should be noted that for spherical particles with constant density *R_g_/R* = 0.78. Thus, one can suppose that the mPEG_113_-*b*-P(D,L)LA*_n_* nanoparticles have higher electron density in the inner part than that in the outer part, i.e., a “core-corona” structure [37,48,51]. It should be noted that the PLA block makes the main contribution to the X-ray scattering due to its higher electron density in comparison with less dense PEG corona. The values of *D_max_/2* and *R* are smaller than the values of *R_h_* or evaluated by DLS (Table 2), which can be explained by higher contribution of the corona.

One can see in Table 2 that the radius of nanoparticles determined both by DSL and SAXS increases with an increase in length of amorphous P(D,L)LA block, which is in accordance with the literature [31,52,53]. Enhanced hydrophobic interactions result in higher aggregation number of the copolymer chains into a nanoparticle leading to an increase in hydrodynamic size [52]. Variation of the polymerization degree of P(D,L)LA block allows us to effectively tune the size of nanoparticles that could be important in optimizing the drug incorporation into these potential drug delivery carriers.

According to the literature the size of obtained mPEG_113_-*b*-P(D,L)LA*_n_* nanoparticles, which is less than 100 nm, could provide delivery of the loaded anticancer agent to tumor in passive targeting manner [30], achieve high tumor extravasation efficacy, and deep tumor penetration of particles regardless of the tumor type [54].

### 2.3. Characterization of Oxaliplatin-Loaded mPEG-b-(D,L)LA Nanoparticles

The values of oxaliplatin loading content in mPEG_113_-*b*-P(D,L)LA*_n_* nanoparticles after removal of free drug are listed in Table 3, which demonstrates that the length of hydrophobic PLA block in mPEG_113_-*b*-P(D,L)LA*_n_* copolymers affects the drug loading. The highest loading content of oxaliplatin was evaluated as 3.8 wt.% for nanoparticles of mPEG_113_-*b*-P(D,L)LA_62_ copolymer with the shortest PLA block. Due to low hydrophobicity of oxaliplatin, its incorporation in the hydrophobic PLA core is unfavorable [12]. We suppose that the anticancer agent could be adsorbed at the core-corona interface of the mPEG_113_-*b*-P(D,L)LA*_n_* nanoparticles due to the highest density of hydrophilic PEG chains there. The size of the nanoparticles decreases with a decrease of PLA block length (Table 3). The smaller size of the mPEG_113_-*b*-P(D,L)LA*_n_* nanoparticles results in enhanced core-corona interface leading to an increase in oxaliplatin loading content.

In order to investigate the influence of oxaliplatin loading on size, morphology, and structure of the mPEG_113_-*b*-P(D,L)LA*_n_* nanoparticles, DLS, TEM, and SAXS were used. Intensity size distribution curves (DLS) for drug-loaded mPEG_113_-*b*-P(D,L)LA_173_ and mPEG_113_-*b*-P(D,L)LA_135_ nanoparticles reveal one peak, except for nanoparticles based on mPEG_113_-*b*-P(D,L)LA_62_ copolymer with the shortest P(D,L)LA block (Figure S2 of the Supplementary Materials). These two peaks could be attributed to individual nanoparticles with small *R_h_* (< 100 nm) and their submicron size aggregates. It should be noted that a fraction of small particles could be undetectable in a mixture with several percent of large particles [47]. In the case of mPEG_113_-*b*-P(D,L)LA_62_, the scattering intensity of individual nanoparticles is high enough to estimate their size (Figure S2 of the Supplementary Materials). Therefore, we suppose that the investigated suspension mainly consists of individual oxaliplatin-loaded nanoparticles with a minor fraction of their aggregates.

The values of *R_h_* of oxaliplatin-loaded mPEG_113_-*b*-P(D,L)LA*_n_* nanoparticles are listed in Table 3. The *R_h_* values of drug-loaded nanoparticles are almost identical (in the limits of experimental uncertainty) to the values of *R_h_* of drug-free nanoparticles (Table 2). Thus, we assume that oxaliplatin loading does not affect the size of the mPEG_113_-*b*-P(D,L)LA*_n_* nanoparticles.

ζ-Potential of oxaliplatin-loaded mPEG_113_-*b*-P(D,L)LA*_n_* nanoparticles was found to be in the range from −16 to −24 mV (Table 3). The values of ζ-potential of drug-loaded nanoparticles are nearly the same as the values for drug-free nanoparticles (Table 2).

The morphology of mPEG_113_-*b*-P(D,L)LA*_n_* nanoparticles remained unchanged with oxaliplatin loading (data not shown).

Scattering curves from oxaliplatin-loaded mPEG_113_-*b*-P(D,L)LA*_n_* nanoparticles in Log *I*–Log *s* coordinates are presented in Figure 4a. All the SAXS profiles converge to a plateau in the region of *s* < 0.1 nm^−1^, indicating the absence of large aggregates. The profiles also show the secondary maximum in the region 0.3 < *s* < 1 nm^−1^ (Figure 4a), suggesting that the nanoparticles have a spherical well-defined structure with a relatively narrow size distribution. As one can see from Figure 3a and Figure 4a, the drug loading does not affect the shape of SAXS curves. Thus, we suggest that oxaliplatin loading does not affect the structure and size of the mPEG_113_-*b*-P(D,L)LA*_n_* nanoparticles.

Figure 4b shows the SAXS curves for both oxaliplatin-loaded and drug-free mPEG_113_-*b*-P(D,L)LA*_n_* nanoparticles in *I·s*^2^–*s* coordinates. As one can see from the Kratky plots (Figure 4b), drug loading leads to an increase of the scattering intensity *I(s)* from the mPEG_113_-*b*-P(D,L)LA*_n_* nanoparticles. This increment could be attributed to a higher average electron density of nanoparticles with incorporated oxaliplatin compared with drug-free nanoparticles.

The *R_g_* values of oxaliplatin-loaded mPEG_113_-*b*-P(D,L)LA*_n_* nanoparticles determined from the Guinier plots (Figure S3 of the Supplementary Materials) are listed in Table 3. It was found that the *R_g_* values of drug-loaded nanoparticles are almost identical (in the limits of experimental uncertainty) to the values of *R_g_* of drug-free nanoparticles (Table 2).

The pair distance distribution functions *P(R)* for oxaliplatin-loaded mPEG_113_-*b*-P(D,L)LA*_n_* nanoparticles are bell-shaped with a well-defined maximum shifted to distances smaller than *D_max_/2* (Figure S4 of the Supplementary Materials). We assume that the shift of the *P(R)* function maximum can be attributed to “core-corona” structure of oxaliplatin-loaded mPEG_113_-*b*-P(D,L)LA*_n_* nanoparticles. The values of *R* and *D_max_/2* corresponding to the peak value and the maximum dimension of the scattering objects are listed in Table 3. *R_g_* values evaluated from *P(R)* functions were compared with *D_max_/2* and *R* values. The *2R_g_/D_max_* values were found to be in the range of 0.61–0.63, whereas *R_g_*/*R* was in the range of 0.80–0.95 (Table 3). Thus, one can suppose that oxaliplatin-loaded mPEG_113_-*b*-P(D,L)LA*_n_* nanoparticles also have higher electron density in the inner part than that in the outer part, i.e., a “core-corona” structure [37,48,51].

Based on SAXS data, we estimated the core-corona interface area of mPEG_113_-*b*-P(D,L)LA*_n_* nanoparticles *s_int_* (Table 4). Nanoparticles of mPEG_113_-*b*-P(D,L)LA_62_ copolymer with the shortest PLA block have the largest core-corona interface area available for oxaliplatin adsorption (Table 4). In addition, as one can see from Table 4, the value of tethering density of hydrophilic PEG chains *σ* on the surface of PLA core is the highest for the mPEG_113_-*b*-P(D,L)LA_62_ particles (detailed description of the calculation can be found in the Supplementary Materials) that is also favorable for oxaliplatin encapsulation. Therefore, mPEG_113_-*b*-P(D,L)LA_62_ particles show the highest values of drug loading content and encapsulation efficacy (Table 4). Based on SAXS data, we suppose that oxaliplatin loading does not affect the size and structure of the mPEG_113_-*b*-P(D,L)LA*_n_* nanoparticles (Table 2 and Table 3). It seems that the amount of loaded drug is insufficient to cause significant changes in these parameters. However, a higher scattering intensity *I(s)* observed for drug-loaded nanoparticles could be attributed to an increase of average electron density of the particles due to oxaliplatin incorporation. Higher initial oxaliplatin loading can lead to a larger drug content in nanoparticles and make it distinguishable on the SAXS curves, which will allow us to investigate the localization of drug in carrier.

Thus, in the present work, we showed that the highest oxaliplatin loading content at the core-corona interface of mPEG_113_-*b*-P(D,L)LA*_n_* nanoparticles through its physical adsorption was 3.8 wt.% with initial drug loading content 5 wt.%. The obtained value is sufficiently higher compared to the value of oxaliplatin loading content in PEG-*b*-PLA nanoparticles reported in ref. [15], i.e., 0.053 wt.%. It should be noted that generally, physical encapsulation of oxaliplatin into polymeric carriers results in lower values of oxaliplatin loading content (3–5 wt.%) [13,26] compared to its content (tens of percent) [14,20,21,22,23,24] after chemical conjugation of oxaliplatin active part with polymer chains. Nevertheless, oxaliplatin-loaded chitosan based polymeric particles showed enhanced cytotoxicity activity against cancer cells compared to free oxaliplatin [26]. Moreover, chitosan-based polymeric micelles and PEG-*b*-PLA nanoparticles with incorporated oxaliplatin are able to selectively accumulate in tumors [15,26].

One of the strategies to enhance the encapsulation efficacy of platinum drugs into polymeric carriers is variation of their lipophilicity. Margiotta et al. investigated the effect of the carboxylate ligand chain length on the encapsulation efficacy of Pt(IV) prodrug complex in poly(lactide-*co*-glycolide)-PEG PLGA-PEG nanoparticles [55]. The authors reported that the amount of Pt atoms encapsulated in PLGA-PEG nanoparticles increased approximately 2 times with an increase of the length of the carboxylate ligand chain from 2 to 10 carbon atoms. In the present work, we studied the effect of mPEG_113_-*b*-P(D,L)LA*_n_* copolymers composition on the encapsulation efficacy of oxaliplatin. Based on the obtained results, we suggest that variation of the ratio of hydrophobic and hydrophilic block lengths in mPEG_113_-*b*-P(D,L)LA*_n_* copolymers can be another useful strategy to enhance Pt-complex loading content in polymeric nanoparticles without chemical modification of the drug (Table 4).

## 3. Materials and Methods

### 3.1. Materials

D,L-lactide (3,6-dimethyl-1,4-dioxane-2,5-dione, 99%) was purchased from Corbion (Netherlands) and recrystallized in butyl acetate before use. Poly(ethylene glycol) methyl ether (mPEG) with a molecular weight of 5000 Da, stannous (II) 2-ethylhexanoate (SnOct_2_) were purchased from Sigma-Aldrich and used as received. All organic solvents were of analytical grade and used without further purification. Double distilled water was used for all experiments. Oxaliplatin (trans-R,R-cyclohexane-1,2-diamine)oxalatoplatinum(II)) was synthesized using a procedure described in literature [56].

### 3.2. Synthesis of Block Copolymers

mPEG_113_-*b*-P(D,L)LA*_n_* diblock copolymers were synthesized by ring-opening polymerization of D,L-lactide in the presence of mPEG. Stannous (II) 2-ethylhexanoate (0.14% wt./wt. with respect to the amount of lactide) was used as a catalyst. By varying the ratio of lactide to mPEG in the reaction, it was possible to control the polymerization degree of PLA block. Before polymerization, the reactants were dried under vacuum for 30 min at room temperature.

For example, for synthesis of mPEG_113_-*b*-P(D,L)LA_62_ block-copolymer, D,L-lactide (5.04 g, 35 mmol), mPEG_113_ (5 g, 1 mmol) were placed into a dried polymerization flask equipped with a magnetic stirrer. Then, 0.017 mL of 1M hexane solution of stannous (II) 2-ethylhexanoate was poured into the flask. Hexane was removed by evaporation in vacuum. The reaction flask was closed with a glass stopper and immersed into an oil bath preheated to 140 °C. The polymerization was carried out for 24 h under argon atmosphere with constant stirring. The reaction product was cooled to room temperature and dissolved in tetrahydrofuran (10 mL) and precipitated twice, first using excess of cold (+5 °C) diethyl ether and then cold hexane (200 mL). The synthesis of block copolymers with different composition was carried out analogously.

### 3.3. Characterization of mPEG-b-P(D,L)LA Copolymers

The degree of lactide conversion, PLA block length and number-average molecular weight of the synthesized polymers were determined by ^1^H NMR. Spectra were recorded on a 300 MHz Bruker WP-250 SY spectrometer in 5 mm o.d. sample tubes. For measurements 30 mg of block copolymer was dissolved in 1 mL of CDCl_3_. The integrals of the peaks corresponding to the PLA methine protons (-CH, 5.15 ppm) and PEG methylene protons (-CH_2_-, 3.65 ppm) were used to calculate PLA block length and number average molecular weight (*M_n_*) of the synthesized block copolymers (Figure S5 of the Supplementary Materials). The degree of conversion was found to be 90–95% for all the synthesized polymers. It was calculated using the integrals of the peaks corresponding to the unreacted lactide and PLA methine protons. The residual monomer was successfully removed by precipitation, as was confirmed by the absence of the corresponding signal (around 4.97–5.05 ppm) on the ^1^H NMR spectrum (Figure S5 of the Supplementary Materials). Thus, the purity of synthesized polymers is not less than 99%.

Gel permeation chromatography (GPC) was performed to determine molecular weight and polydispersity index of the synthesized block copolymers (Figure S6 of the Supplementary Materials). Chromatograms were recorded on a Knauer system consisting of a pump, a refractometric detector, and Phenogel 5 µm 103 Å column. The sample concentration was 5 g/L, THF was used as the mobile phase (40 °C and 1 mL/min), and the column calibration was performed with polystyrene standards (Polymer Laboratories).

### 3.4. Preparation of mPEG-b-P(D,L)LA Nanoparticles

Drug-free and drug-loaded nanoparticles based on mPEG-*b*-P(D,L)LA copolymers were prepared by solvent displacement (nanoprecipitation) method with acetone as organic solvent [57]. Briefly, mPEG-*b*-P(D,L)LA (50 mg) was dissolved in acetone (10 mL). Double distilled water (10 mL) was added dropwise into the solution under stirring. The organic solvent was removed through evaporation for 4 h at room temperature.

To prepare oxaliplatin-loaded mPEG-*b*-P(D,L)LA nanoparticles, 2.5 mg of oxaliplatin (5% wt./wt. with respect to the amount of block copolymer) was preliminarily dissolved in water. Then, the nanoparticles were prepared similarly to the drug-free mPEG-*b*-P(D,L)LA nanoparticles. Finally, the aqueous suspensions were centrifuged (40000 g, 30 min) to remove the residues of the organic solvent and free drug, and the precipitated nanoparticles were dispersed in water and freeze-dried.

### 3.5. Characterization of mPEG-b-P(D,L)LA Nanoparticles

Measurements of size distribution and zeta-potential (ζ-potential) were performed by dynamic light scattering (DLS) on a Zetasizer Nano ZS instrument (Malvern Ltd.), equipped with a He-Ne laser with a wavelength of 633 nm at a scattering angle of 173°. All of the experiments were carried out three times. The data are presented as mean ± standard deviation.

The morphology of nanoparticles was characterized by transmission electron microscopy (TEM) using a Titan 80–300 TEM/STEM (FEI) microscope at accelerating voltage of 300 kV with a BM-Ultrascan (Gatan) camera operating in the bright field mode. Thin-carbon-film-coated copper TEM grids were glow-discharged for 10 s in the Pelco easiGlow system. A 3 μL droplet of the aqueous suspensions with concentration of 0.5 g/L was deposited on the carbon side of the grid and incubated for 1 min. Then, the carbon side of the grid was rinsed with 10 μL of distilled water, and right after that, 10 μL of uranyl acetate solution with a concentration of 1 wt.% was applied to the grid and incubated for 30 s. The excess of the solution was removed after each step by touching the grid edge with filter paper.

Synchrotron SAXS measurements of the aqueous suspensions of nanoparticles were performed at the European Molecular Biology Laboratory (EMBL) on the storage ring PETRA III (DESY, Hamburg) on the EMBL-P12 beamline equipped with a 2D photon counting pixel X-ray detector Pilatus 2 M (Dectris). The scattering intensity, *I(s)*, was recorded in the range of the momentum transfer 0.02 < *s* < 4.5 nm^−1^, where

s = (4π sin θ)/λ(1)

*2θ* is the scattering angle, and λ = 0.124 nm is the X-ray wavelength. The measurements were carried out at 23 °C using continuous flow operation over a total exposure time of 1 s divided into 20 × 50 ms individual frames to monitor for potential radiation damage (no radiation effects were detected). For each sample, 20 scattering curves were captured to improve the quality of the obtained data. The data were corrected for the solvent scattering and processed using standard procedures with the program PRIMUS [58]. Data analysis was performed using the software suite ATSAS [59]. Pair distance distribution and volume size distribution functions were calculated using the program GNOM [60]. Before SAXS measurements, all suspensions were centrifuged (10000 rpm, 10 min).

### 3.6. Evaluation of Drug Loading

The content of oxaliplatin (weight ratio of the drug to the block copolymer) in the freeze-dried nanoparticles was determined by inductively coupled plasma atomic emission spectroscopy (ICP-AES). An axial ICP-AES 720-ES spectrometer (Agilent Technologies, USA) was used for measurements with a low flow axial quartz torch with 2.4 mm inner diameter injector tube (Glass Expansion, Australia), a double-pass glass cyclonic spray chamber (Agilent Technologies), a OneNeb nebulizer (Agilent Technologies, USA), and a Trident Internal Standard Kit (Glass Expansion). Samples were introduced manually to reduce washing volume, without preliminary digestion or dilution. A detailed description of the measurement process can be found in the Supplementary Materials. The drug loading content DLC and encapsulation efficacy EE of oxaliplatin-loaded mPEG-*b*-P(D,L)LA nanoparticles were calculated according to the following equations:

(2) $$DLC=\;\frac{m_{1}^{OxPt}}{m_{NP}}\times 100\%$$

(3) $$EE=\;\frac{m_{1}^{OxPt}}{m_{o}^{OxPt}}\times 100\%$$

where $m_{1}^{OxPt}$ is amount of incorporated oxaliplatin in nanoparticles, $m_{NP}$ is amount of nanoparticles, and $m_{0}^{OxPt}$ is initial amount of oxaliplatin.

## 4. Conclusions

Drug-free and oxaliplatin-loaded biodegradable mPEG_113_-*b*-P(D,L)LA*_n_* nanoparticles were prepared by a simple nanoprecipitation technique. The influence of hydrophobic block length on the structure, size, morphology, and drug loading content of mPEG_113_-*b*-P(D,L)LA*_n_* nanoparticles was investigated. It was observed that in aqueous solution mPEG_113_-*b*-P(D,L)LA*_n_* copolymers, where *n* = 62–173 monomer units, form spherical nanoparticles with hydrodynamic diameters ranging from 32 to 56 nm. The “core-corona” structure of the block copolymer nanoparticles was confirmed by SAXS. Tailoring of P(D,L)LA block length results in variation in both core-corona interface area and tethering density of hydrophilic PEG chains on the surface of P(D,L)LA core of the mPEG_113_-*b*-P(D,L)LA*_n_* nanoparticles, which affects oxaliplatin loading content. An increase in P(D,L)LA block length from 62 to 173 monomer units results in a decrease in core-corona interface area from 2.7 × 10^20^ to 1.5 × 10^20^ nm^2^/g and tethering density of PEG chains from 1.6 to 1.0 nm^−2^ and a reduction in the oxaliplatin loading content from 3.8 to 1.5% wt./wt. Thus, we suppose that oxaliplatin is adsorbed on the core-corona interface of the mPEG_113_-*b*-P(D,L)LA*_n_* nanoparticles. SAXS measurements revealed that oxaliplatin loading does not affect the size and structure of the block copolymer nanoparticles.

The size and structure of polymeric nanoparticles are crucial characteristics that should be considered in the design of targeted nanoformulations of anticancer agents. The developed oxaliplatin formulation based on 32 nm mPEG_113_-*b*-P(D,L)LA*_62_* nanoparticles loaded with 3.8 wt.% of drug with 76% encapsulation efficiency can be considered as a promising candidate for treatment of various types of cancer. In vitro and in vivo tests will be performed in order to compare its efficacy and toxicological profile with pure oxaliplatin.

## Figures and Tables

**Table 1:** Molecular characteristics of the synthesized diblock copolymers

Sample	*M_n_*^1^, g/mol	*M_n_*^2^, g/mol	*M_w_*^2^, g/mol	PDI ^2^
mPEG_113_-*b*-P(D,L)LA_62_	9500	7300	10,500	1.4
mPEG_113_-*b*-P(D,L)LA_135_	14,700	9000	14,600	1.6
mPEG_113_-*b*-P(D,L)LA_173_	17,500	10,400	18,000	1.7

^1^ Determined by ^1^H NMR. ^2^ Determined by GPC.

**Table 2:** Characteristics of the mPEG_113_-*b*-P(D,L)LA*_n_* nanoparticles in aqueous suspensions.

Sample	*R_h_*^1^, nm	*R_g_*^2^, nm	*R_g_*^3^, nm	*D_max_/2*^4^, nm	*R*^5^, nm	*2R_g_/D_max_*	*R_g_/R*	ζ ^6^, mV
mPEG_113_-*b*- P(D,L)LA_173_	28 ± 10	11.4 ± 0.1	12.5 ± 0.1	19.5 ± 1	15.4 ± 1	0.64 ± 0.05	0.81 ± 0.06	−14 ± 8
mPEG_113_-*b*- P(D,L)LA_135_	22 ± 10	10.1 ± 0.1	9.7 ± 0.1	17 ± 1	11.5 ± 1	0.57 ± 0.06	0.84 ± 0.09	−15 ± 4
mPEG_113_-*b*- P(D,L)LA_62_	16 ± 6	9.1 ± 0.1	8.6 ± 0.1	14 ± 1	9 ± 1	0.61 ± 0.07	0.96 ± 0.11	−20 ± 4

^1^ The value of *R_h_* corresponding to the peak position on DLS intensity size distribution curve. ^2^ Gyration radius of nanoparticles calculated from Guinier plots. ^3^ Gyration radius of nanoparticles evaluated from *P(R)*. ^4^ Radius of nanoparticles evaluated from *P(R)*. ^5^ The value of *R* corresponding to the maximum position on *P(R)* function. ^6^ ζ-potential of nanoparticles.

**Table 3:** Characteristics of oxaliplatin-loaded mPEG_113_-*b*-P(D,L)LA*_n_* nanoparticles in aqueous suspensions.

Sample	*R_h_*^1^, nm	*R_g_*^2^, nm	*R_g_*^3^, nm	*D_max_/2*^4^, nm	*R*^5^, nm	*2R_g_/D_max_*	*R_g_/R*	ζ ^6^, mV	DLC ^7^, wt.%
mPEG_113_-*b*- P(D,L)LA_173_	27 ± 10	11.1 ± 0.1	12.3 ± 0.1	19.5 ± 1.0	15.3 ± 1.0	0.63 ± 0.05	0.80 ± 0.07	−16 ± 9	1.5
mPEG_113_-*b*- P(D,L)LA_135_	25 ± 12	9.4 ± 0.1	10.4 ± 0.1	17.0 ± 1.0	12.2 ± 1.0	0.61 ± 0.06	0.85 ± 0.08	−17 ± 4	2.3
mPEG_113_-*b*- P(D,L)LA_62_	19 ± 8	9.3 ± 0.1	8.7 ± 0.1	14.0 ± 1.0	9.2 ± 1.0	0.62 ± 0.07	0.95 ± 0.11	−24 ± 8	3.8

^1^ The value of *R_h_* corresponding to the peak position on DLS intensity size distribution curve. ^2^ Gyration radius of nanoparticles calculated from Guinier plots. ^3^ Gyration radius of nanoparticles evaluated from *P(R)*. ^4^ Radius of nanoparticles evaluated from *P(R)*. ^5^ The value of *R* corresponding to the maximum position on *P(R)* function. ^6^ ζ-potential of nanoparticles. ^7^ Drug loading content determined by ICP-AES.

**Table 4:** Parameters of oxaliplatin-loaded mPEG_113_-*b*-P(D,L)LA*_n_* nanoparticles.

Sample	*R_c_*^1^, nm	*s_int_*^2^, nm^2^/g	*σ*^3^,nm^−2^	DLC ^4^, wt.%	EE ^5^, %
mPEG_113_-*b*-P(D,L)LA_173_	15.3	1.5 × 10^20^	1.0	1.5	30
mPEG_113_-*b*-P(D,L)LA_135_	12.2	2.0 × 10^20^	0.9	2.3	46
mPEG_113_-*b*-P(D,L)LA_62_	9.2	2.7 × 10^20^	1.6	3.8	76

^1^ The value of PLA core radius evaluated from SAXS (equivalent to the maximum position *R* on the pair distance distribution function *P(R)*). ^2^ The value of core-corona interface area *s_int_*. ^3^ The value of tethering density of PEG chains *σ* on the PLA core surface. ^4^ The value of oxaliplatin loading content. ^5^ The value of encapsulation efficacy.

## Data Availability

The data presented in this study are available on request from the corresponding author.

## Supplementary Materials

The following are available online, Figure S1: Guinier plots for the mPEG_113_-*b*-P(D,L)LA*_n_* nanoparticles, Figure S2: DLS intensity size distribution curves for oxaliplatin-loaded mPEG_113_-*b*-P(D,L)LA*_n_* nanoparticles, Figure S3: Guinier plots for oxaliplatin-loaded mPEG_113_-*b*-P(D,L)LA*_n_* nanoparticles, Figure S4: Pair distance distribution functions *P(R)*–*R* for oxaliplatin-loaded mPEG_113_-*b*-P(D,L)LA*_n_* nanoparticles, Figure S5: Typical ^1^H NMR spectrum of mPEG_113_-*b*-P(D,L)LA*_n_* (300 MHz, CDCl_3_), Figure S6: GPC curves for synthesized mPEG_113_-*b*-P(D,L)LA*_n_* copolymers, Table S1: Conditions of ICP-AEC measurements.

## Sample Availability

Samples of the compounds are not available from the authors.
