# Supplementary material for: Poly(Ethylene Glycol)-b-Poly(D,L-Lactide) Nanoparticles as Potential Carriers for Anticancer Drug Oxaliplatin
Source: Molecules. 2021 Jan 24;26(3):602. doi: 10.3390/molecules26030602 (PMC7865450; doi:10.3390/molecules26030602)
Supplement: Supplementary file 1 [file molecules-26-00602-s001.pdf]

Supplementary Materials

Figure S1. Guinier plots for the mPEG$_{113}$-*b*-P(D,L)LA$_n$ nanoparticles: 1 – mPEG$_{113}$-*b*-P(D,L)LA$_{173}$ (c = 7,5 g/L), 2 – mPEG$_{113}$-*b*-P(D,L)LA$_{135}$ (c = 5 g/L), 3 – mPEG$_{113}$-*b*-P(D,L)LA$_{62}$ (c = 5 g/L). The curves are shifted vertically for clarity.

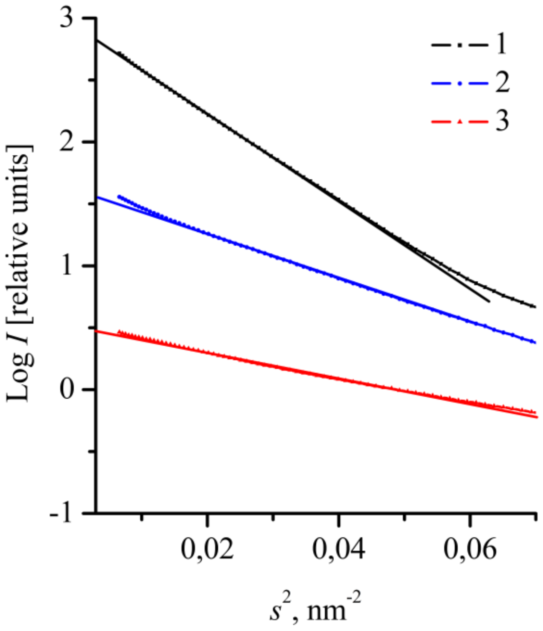

Figure S2 DLS intensity size distribution curves for oxaliplatin-loaded mPEG$_{113}$-*b*-P(D,L)LA$_n$ nanoparticles (c = 0.5 g/L): 1 – mPEG$_{113}$-*b*-P(D,L)LA$_{173}$, 2 – mPEG$_{113}$-*b*-P(D,L)LA$_{135}$, 3 – mPEG$_{113}$-*b*-P(D,L)LA$_{62}$.

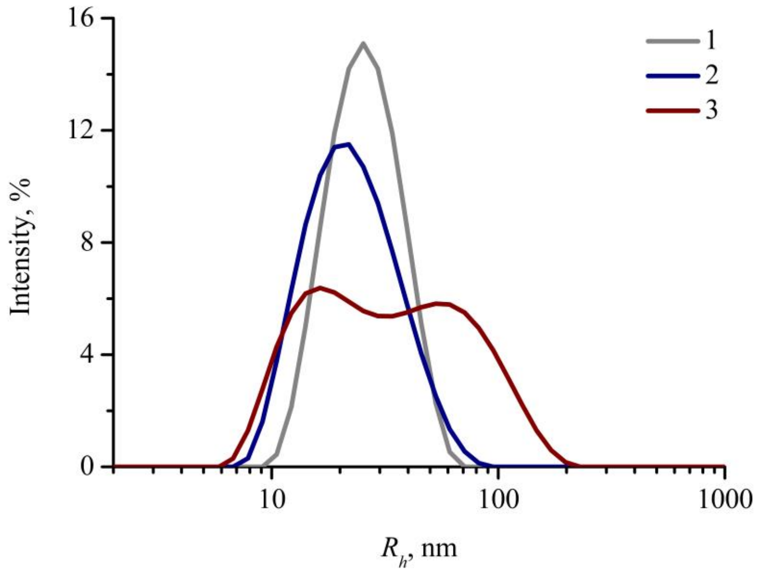

Figure S3. Guinier plots for oxaliplatin-loaded mPEG$_{113}$-*b*-P(D,L)LA$_n$ nanoparticles: 1 – mPEG$_{113}$-*b*-P(D,L)LA$_{173}$ (c = 7,5 g/L), 2 – mPEG$_{113}$-*b*-P(D,L)LA$_{135}$ (c = 5 g/L), 3 – mPEG$_{113}$-*b*-P(D,L)LA$_{62}$ (c = 5 g/L). The curves are shifted vertically for clarity.

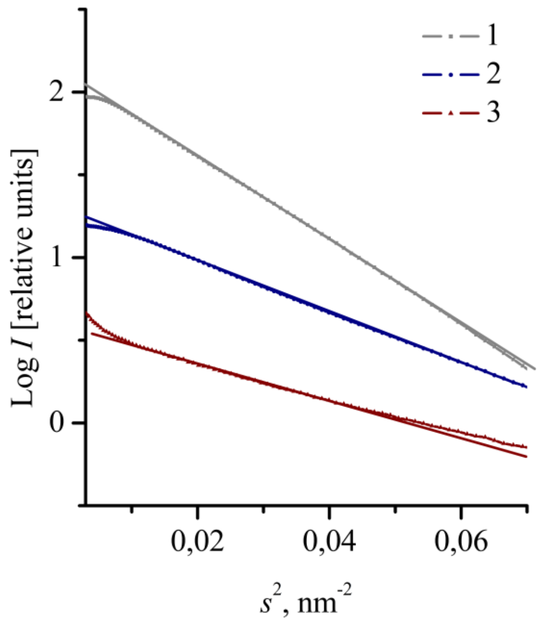

Figure S4. Pair distance distribution functions *P(R)* – *R* for oxaliplatin-loaded mPEG$_{113}$-*b*-P(D,L)LA$_n$ nanoparticles: 1 – mPEG$_{113}$-*b*-P(D,L)LA$_{173}$ (c = 7,5 g/L), 2 – mPEG$_{113}$-*b*-P(D,L)LA$_{135}$ (c = 5 g/L), 3 – mPEG$_{113}$-*b*-P(D,L)LA$_{62}$ (c = 5 g/L).

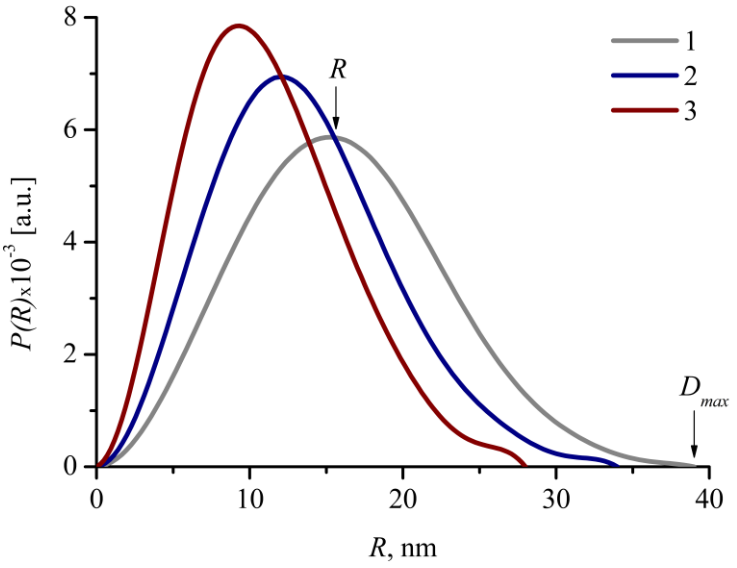

Evaluation of parameters of oxaliplatin-loaded $mPEG_{113}$-b-P(D,L)LA_n nanoparticles.

The values of core-corona interface area s_{int} of the $mPEG_{113}$-*b*-P(D,L)LA_n nanoparticles were calculated from the radius of poly(lactide) PLA core R_c determined by SAXS according to the following equation:

$$s_{int} = \frac{3}{\rho \cdot R_c},$$

where ρ = 1.25 g/cm^3 is the bulk density of PLA.

The values of tethering density of hydrophilic PEG chains σ on the PLA core surface of the $mPEG_{113}$-*b*-P(D,L)LA_n nanoparticles were calculated according to the equation:

$$\sigma = \frac{1}{s} = \frac{N_{agg}}{4\pi R_c^2},$$

where s is the PLA core area corresponding to one PEG chain, N_{agg} is the aggregation number of nanoparticle.

The values of N_{agg} of the $mPEG_{113}$-*b*-P(D,L)LA_n nanoparticles were calculated according to the following equation:

$$N_{agg} = \frac{R_c^3 4\pi N_A \rho}{3 M_o N_{PLA} 10^{21}},$$

where N_A is the Avogadro constant, M_o is the molecular weight of PLA monomer unit, N_{PLA} is the polymerization degree of PLA block.

Figure S5. Typical ^{1}H NMR spectrum of mPEG$_{113}$-*b*-P(D,L)LA$_n$ (300 MHz, CDCl3): δ = 5.07-5.28 (m., H1), δ = 4.34 (q., H2), δ = 4.28 (tr., H3), δ = 3.64 (s., H4), δ = 3.36 (s., H5), 1.50-1.64 (m., H6), 1.47 (d., H7).

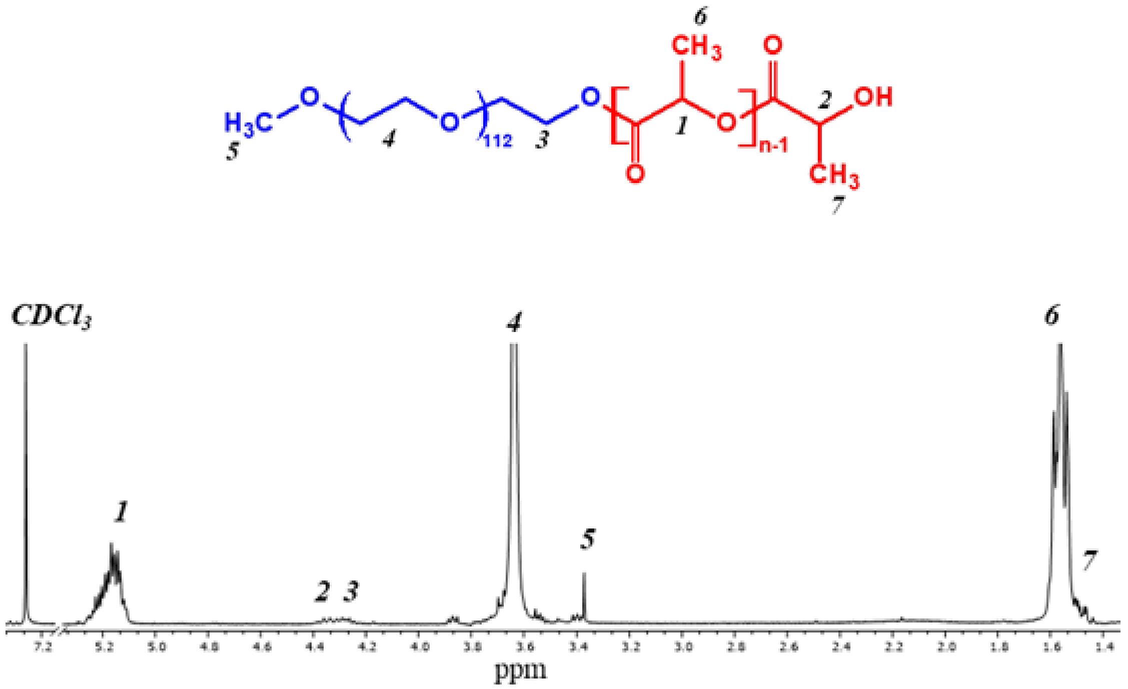

Figure S6. GPC curves for synthesized mPEG$_{113}$-*b*-P(D,L)LA$_n$ copolymers: 1 – mPEG$_{113}$-*b*-P(D,L)LA$_{173}$, 2 – mPEG$_{113}$-*b*-P(D,L)LA$_{135}$, 3 – mPEG$_{113}$-*b*-P(D,L)LA$_{62}$.

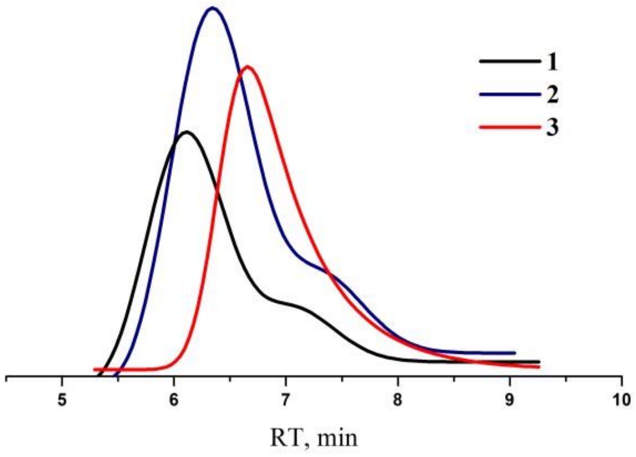

Evaluation of drug loading (ICP-AES analysis).

Sample digestion.

A microwave system Ultraclave III (Milestone GMBH) was used for sample acid digestion. A sample weight taken with an accuracy of 0.1 mg was placed in quartz digestion tube of Ultraclave III system, than 5 ml of HNO_3 (69%, ACS-ISO grade, Panreac) were added. Tubes were heated up to 250 °C, hold during 30 min at 250 °C and pressure 120 bar, than cooled. After depressurization solutions were transferred to a 10 ml polypropylene volumetric flasks, diluted with deionized water and analyzed.

ICP-AES analysis.

An axial ICP-AES 720-ES spectrometer (Agilent Technologies, USA) was used for measurements with a low flow axial quartz torch with 2.4 mm inner diameter injector tube (Glass Expansion, Australia), a double-pass glass cyclonic spray chamber (Agilent Technologies), a OneNeb nebulizer (Agilent Technologies, USA), and a Trident Internal Standard Kit (Glass Expansion). Samples were introduced manually to reduce washing volume, without preliminary digestion or dilution. A peristaltic pump used the white/white poly-vinyl chloride pump tube for feeding and the blue/blue one for drain. A Sc solution (20 ppm) internal standard was added online (orange/blue poly-vinyl chloride pump tube) to increase the accuracy of measurements. Conditions of ICP–AES measurements are presented in Table S1. Results were collected and processed by ICP Expert software 2.0.5 (Agilent Technologies). All lines were measured simultaneously (a MultiCal mode). Linear and rational (quadratic) functions were used for the calibration. The position and baseline correction were made for all the peaks of uncommon elements using the tools of the spectrometer software.

Table S1. Conditions of ICP–AES measurements.

**Conditions for all lines registrations**	
RF power (kW)	1.40
Plasma flow (L/min)	18.0
Axial flow (L/min)	1.50
Nebulizer flow (L/min)	1.00
Replicate read time (s)	20
Instrument stabilization delay (s)	15
Replicates	3
**Sample introduction settings**	
Sample uptake delay (s)	25
Pump rate (rpm)	12

Deionized water (18.2 MΩ×cm from a Milli-Q Academic system, Millipore, France) was used for the preparation of all the solutions and washing. A standard solution of platinum, 1000 mg/L (High Purity Standards) was used for calibration in the range 0.01 – 10 mg/L. An internal standard solution of scandium (20 mg/L) was prepared from Sc standard solution, 1000 mg/L (High Purity Standards). Emission lines used for ICP–AES measurements were 214.424; 217.468; 224.552; 265.945; 273.396 and 306.471 nm for platinum and 361.383 nm for scandium. The measurement results obtained on different lines were averaged with differences between them less than 5% and were used for final calculations.
